# Evaluation of diverse polygenic risk score models for lung cancer in a small-scale Chinese cohort

**DOI:** 10.3389/fgene.2025.1646997

**Published:** 2025-07-16

**Authors:** Min Gao, Qiwen Zheng, Yue Jiang, Xiao Chang, Xin Zheng

**Affiliations:** ^1^ The First College of Clinical Medicine, Shandong University of Traditional Chinese Medicine, Jinan, Shandong, China; ^2^ Department of Radiotherapy, Shandong Cancer Hospital and Institute, Shandong First Medical University and Shandong Academy of Medical Sciences, Jinan, Shandong, China; ^3^ Department of Internal Medicine, Qingdao Hiser Hospital Affiliated of Qingdao University (Qingdao Traditional Chinese Medicine Hospital), Qingdao, Shandong, China; ^4^ China National Center for Bioinformation, Chinese Academy of Sciences, Beijing, China; ^5^ Beijing Institute of Genomics, Chinese Academy of Sciences, Beijing, China; ^6^ College of Medical Information and Artificial Intelligence, Shandong First Medical University, Jinan, Shandong, China; ^7^ The Department of Otorhinolaryngology Head and Neck Surgery, Yantai Yuhuangding Hospital, Qingdao University, Qingdao, Shandong, China

**Keywords:** polygenic risk score, lung cancer, PRS-CS, Chinese population, genome-wide association study

## Abstract

**Introduction:**

Lung cancer is a leading cause of cancer-related mortality globally, with distinct epidemiological and genetic patterns in East Asian populations. However, most polygenic risk score (PRS) models have been developed using European-ancestry cohorts, raising concerns about their applicability in non-European populations.

**Materials and methods:**

In this study, we systematically evaluated the predictive performance of three PRS approaches in a Chinese lung cancer cohort consisting of 97 cases and 667 controls. We assessed (i) a previously reported 19-SNP PRS developed in Chinese individuals, (ii) genome-wide PRS derived using PRS-CS with East Asian and European GWAS summary statistics, and (iii) PRS-CSx, a cross-population Bayesian framework that integrates summary statistics across ancestries.

**Results:**

The 19-SNP PRS demonstrated limited discriminative power in our cohort. In contrast, PRS-CS using East Asian summary statistics showed significant associations with overall lung cancer and specific histological subtypes, particularly NSCLC and LUAD. PRS-CS based on European data yielded weaker performance, underscoring the importance of ancestry matching. Notably, PRS-CSx outperformed single-ancestry models, achieving improved risk stratification for NSCLC and LUAD. However, its predictive performance for LUSC and SCLC remained limited, likely due to sample size constraints and subtype heterogeneity.

**Conclusion:**

Our findings emphasize the critical role of ancestry-matched data and integrative PRS approaches in enhancing risk prediction in underrepresented populations. PRS-CSx represents a promising tool for lung cancer risk assessment in East Asians, though further validation in larger cohorts are needed to improve generalizability and clinical utility.

## 1 Introduction

Lung cancer is a leading cause of cancer-related death globally, with both genetic and environmental factors contributing to its etiology (Bray et al., 2018). Genome-wide association studies (GWAS) have identified multiple genetic variants associated with lung cancer risk, enabling the development of polygenic risk scores (PRS) that aggregate individual variant effects to estimate genetic susceptibility (Dai et al., 2019; Lebrett et al., 2023; Wang et al., 2024; Boumtje et al., 2024; Ma et al., 2025). PRS have shown promise in improving risk prediction and stratification, which can facilitate personalized prevention and early detection.

However, most lung cancer GWAS and PRS models have been developed primarily in European populations (Lebrett et al., 2023; Wang et al., 2024; Boumtje et al., 2024), limiting their applicability to other ancestries due to differences in allele frequencies, linkage disequilibrium patterns, and genetic architecture. East Asian populations, including the Chinese, remain underrepresented in genetic risk prediction studies, and the transferability of existing PRS models to these populations is uncertain. Moreover, while PRS methods are typically validated in large cohorts, In real-world settings many studies have relatively small sample sizes, posing challenges for model performance and validation.

In this study, we utilized a Chinese lung cancer cohort comprising 97 cases and 667 cancer-free controls to systematically evaluate different PRS construction strategies. We implemented PRS-CS and PRS-CSx, state-of-the-art Bayesian regression frameworks that leverage continuous shrinkage priors for improved effect size estimation and allow multi-ancestry summary statistics integration (Ge et al., 2019; Ruan et al., 2022). Specifically, PRS-CSx jointly models GWAS summary statistics from multiple populations to enhance cross-ancestry prediction accuracy. In addition, we examined a previously published 19-SNP PRS developed for Chinese populations (Ruan et al., 2022) as a benchmark for comparison.

By comparing PRS models constructed from Asian-only GWAS, combined Asian-European GWAS, and the 19-SNP PRS in our modestly sized cohort, we aimed to assess their predictive performance and robustness under limited sample size conditions. This evaluation provides critical insights into the applicability of diverse PRS approaches for lung cancer risk prediction in Chinese populations and guides future efforts to develop equitable and accurate genetic risk models for precision oncology.

## 2 Methods

### 2.1 Participants

A total of 97 lung cancer cases were recruited from the Shandong Cancer Hospital and Institute. All patients were ethnic Han Chinese adults diagnosed with lung cancer and either receiving treatment or undergoing surgery at the facility. Peripheral blood samples were obtained at diagnosis, and clinical information such as age, tumor location, and staging was recorded by the attending physicians. A summary of key demographic and clinical characteristics of the patient cohort is provided in Supplementary Table S1. The control group consisted of 667 individuals drawn from the CAS cohort, a prospective multi-omics study involving adult participants from various institutions affiliated with the Chinese Academy of Sciences in Beijing. Participants with any self-reported history of cancer were excluded based on questionnaire data. Written informed consent was secured from all participants or their legal representatives. The study protocol received ethical approval from the Institutional Review Boards of the Shandong Cancer Hospital and Institute, the Beijing Institute of Genomics (Chinese Academy of Sciences), and Beijing Zhongguancun Hospital.

### 2.2 Genotyping, quality control (QC) and imputation

Genotyping was performed using the Infinium Asian Screening Array (ASA), while samples in the replication cohort were genotyped with either the Illumina HumanHap550 or HumanHap610 SNP arrays. Samples with a genotype call rate below 95% were excluded to ensure data quality. Single nucleotide polymorphisms (SNPs) were filtered out if they exhibited a minor allele frequency (MAF) less than 1%, a call rate below 98%, or significant deviation from Hardy–Weinberg equilibrium (P < 1 × 10^−6^). Genotype imputation was conducted via the TOPMed Imputation Server using the minimac4 algorithm, referencing the TOPMed panel composed of whole-genome sequencing data from over 100,000 individuals. For subsequent association analyses, only imputed variants with MAF greater than 1% and imputation quality score (Rsq) exceeding 0.5 were retained to ensure reliability.

### 2.3 PRS construction

The GWAS summary statistics used for polygenic risk score construction in this study were obtained from the GWAS Catalog, focusing on lung cancer association results relevant to Asian and European populations. Detailed information on the included GWAS datasets, including sample sizes, ancestry composition, and reference sources, is provided in Supplementary Table S2.

To estimate posterior SNP effect sizes for PRS construction, we applied two advanced Bayesian polygenic prediction methods: PRS-CS and PRS-CSx (Ge et al., 2019; Ruan et al., 2022). PRS-CS utilizes continuous shrinkage priors to model linkage disequilibrium patterns within a single ancestry GWAS summary, improving effect size estimation and prediction accuracy. PRS-CSx extends this framework to jointly model GWAS summary statistics across multiple ancestries, enabling better cross-population polygenic risk prediction by leveraging shared and population-specific genetic architectures. PRS-CS/PRS-CSx generates posterior SNP effect size estimates separately for each chromosome. Individual-level polygenic risk scores were then calculated by concatenating these per-chromosome files and applying PLINK’s--score command. When scoring was performed chromosome-wise, the ‘sum’ modifier was used to aggregate scores into a genome-wide polygenic score. For PRS-CSx, ancestry-specific posterior SNP effect sizes were first estimated separately for the East Asian and European GWAS datasets. To construct the final polygenic risk score, we computed a linear combination of the ancestry-specific PRS using empirically determined mixing weights. These weights were selected through 10-fold cross-validation within the target dataset, as recommended by the original PRS-CSx framework. No manually assigned ancestry weights or tuning parameters were used. This data-driven approach allows the model to optimize the relative contributions of each ancestry based on their predictive performance, thereby enhancing cross-ancestry applicability. In addition to these methods, we evaluated a previously published 19-SNP PRS model developed specifically for the Chinese population. This panel has been validated in Chinese cohorts and served as a benchmark for comparison against PRS-CS and PRS-CSx derived scores.

## 3 Results

### 3.1 Evaluation of the 19-SNP polygenic risk score in the Chinese lung cancer cohort

**FIGURE 1:**
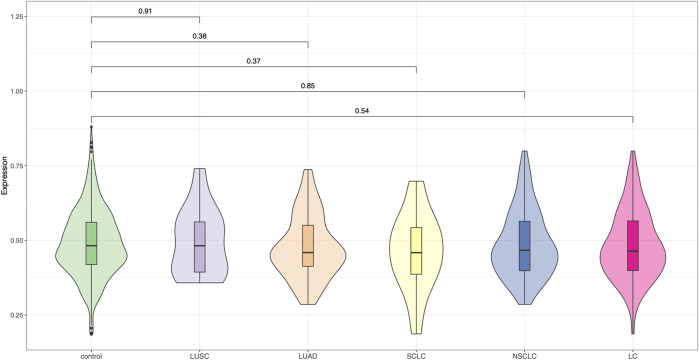
Evaluation of the 19-SNP polygenic risk score in a Chinese lung cancer cohort. Violin plots show the distribution of PRS values across control individuals and five lung cancer subgroups: LUSC (lung squamous cell carcinoma), LUAD (lung adenocarcinoma), SCLC (small-cell lung cancer), NSCLC (non-small-cell lung cancer), and overall lung cancer (LC). The 19-SNP PRS, originally developed for Chinese populations, did not demonstrate significant discrimination between cases and controls in this cohort, suggesting limited predictive utility in this small sample.

We evaluated the performance of a previously established 19-SNP polygenic risk score (PRS), developed for Chinese populations, in our independent Chinese lung cancer cohort. Across all lung cancer cases as well as within major histological subtypes—including lung adenocarcinoma (LUAD), lung squamous cell carcinoma (LUSC), small cell lung cancer (SCLC), and non-small cell lung cancer (NSCLC)—the PRS showed no statistically significant difference between cases and controls (all *P*-values >0.05, Figure 1). There was no clear improvement in risk discrimination for any specific subtype. These findings indicate that the 19-SNP PRS did not exhibit meaningful predictive performance in our sample and may have limited generalizability to independent Chinese cohorts. Further refinement and validation in larger and more diverse populations are warranted.

### 3.2 Evaluation of PRS-CS–derived polygenic risk score in the Chinese lung cancer cohort

**FIGURE 2:**
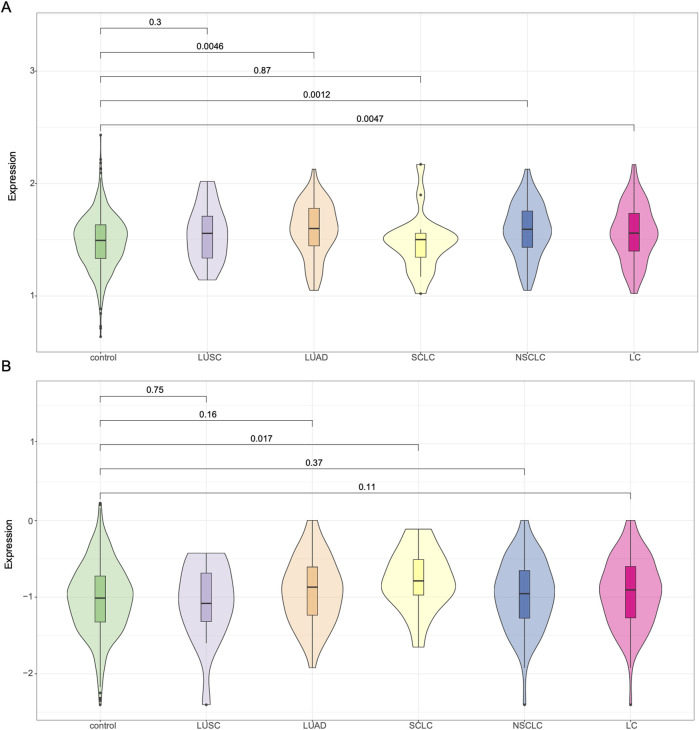
Violin plots of polygenic risk scores generated using PRS-CS. **(A)** PRS constructed using East Asian GWAS summary statistics. **(B)** PRS constructed using European GWAS summary statistics. PRS distributions are shown for control individuals and major lung cancer subtypes: LUSC, LUAD, SCLC, NSCLC, and overall lung cancer (LC).

In addition to evaluating the previously established 19-SNP polygenic risk score (PRS), we applied the PRS-CS method to generate genome-wide PRS using GWAS summary statistics derived from both East Asian and European populations (Figure 2). When using East Asian GWAS summary statistics, the PRS-CS model showed statistically significant differences between lung cancer cases and controls (P = 0.0047). Subtype-specific analysis revealed significant differences for NSCLC (*P* = 0.0012) and LUAD (*P* = 0.0046), while lung squamous cell carcinoma (LUSC) cases also had elevated PRS compared to controls, though the difference did not reach significance (*P* = 0.30). No appreciable difference was observed in the small cell lung cancer (SCLC) subgroup. We then repeated the analysis using PRS-CS based on European GWAS summary statistics. In this setting, lung cancer cases exhibited a trend toward higher PRS than controls (*P* = 0.11), but the difference was not statistically significant overall. Interestingly, among the subtypes, only SCLC reached nominal significance (*P* = 0.017), whereas no clear separation was observed for NSCLC, LUAD, or LUSC. Taken together, these results suggest that PRS-CS models based on East Asian GWAS summary statistics perform better in our Chinese lung cancer cohort than those derived from European data, particularly for NSCLC and LUAD. Nonetheless, the limited performance in certain subtypes highlights the need for larger ancestry-matched GWAS and refined PRS models for improved risk prediction.

### 3.3 Evaluation of PRS-CSx–derived polygenic risk score in the Chinese lung cancer cohort

**FIGURE 3:**
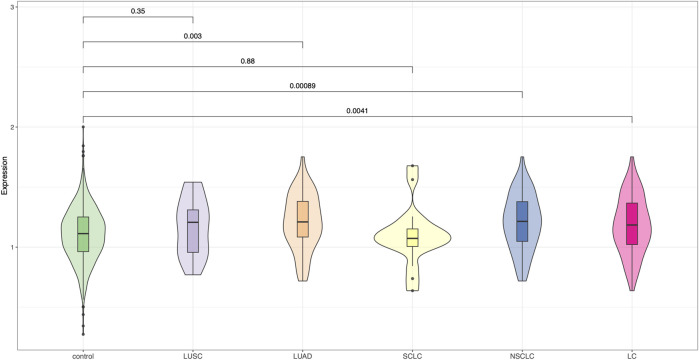
Violin plots of polygenic risk scores generated using PRS-CSx. PRS distributions are shown across control individuals and major lung cancer subtypes: LUSC, LUAD, SCLC, NSCLC, and overall lung cancer (LC), using PRS-CSx models based on East Asian and European GWAS data.

We further applied PRS-CSx, a Bayesian polygenic modeling framework that integrates GWAS summary statistics from multiple ancestral populations, to improve polygenic prediction in the Chinese lung cancer cohort. Using East Asian–derived GWAS as the primary population and incorporating cross-ancestry information from European GWAS, the PRS-CSx–derived risk scores demonstrated improved discriminative performance compared to the 19-SNP PRS and single-ancestry PRS-CS models (Figure 3). Notably, the PRS scores were significantly higher in overall lung cancer cases than in controls (*P* = 0.0041), with the strongest signal observed in the NSCLC subgroup (*P* = 0.00089), followed by LUAD (*P* = 0.003). Although LUSC showed a trend toward elevated PRS compared to controls, the association was not statistically significant (*P* = 0.35). In contrast, no meaningful separation was detected in the SCLC group. These findings suggest that PRS-CSx effectively improves polygenic risk prediction for certain histologic subtypes, particularly NSCLC and LUAD, within East Asian populations by leveraging cross-population genetic architecture.

## 4 Discussion

In this study, we systematically evaluated the predictive performance of various polygenic risk score (PRS) models for lung cancer in a Chinese cohort comprising 97 cases and 667 controls. Our investigation compared three strategies: a previously published 19-SNP PRS designed for Chinese populations, PRS-CS derived from ancestry-matched or European GWAS, and PRS-CSx, a cross-ancestry Bayesian method that integrates summary statistics from multiple populations. Through these comparisons, we aimed to assess the utility and limitations of current PRS methodologies in East Asian populations with relatively small sample sizes.

The 19-SNP PRS, although developed for Chinese individuals, did not yield statistically significant risk stratification in our cohort, underscoring its limited generalizability and the need for continual model refinement. By contrast, the genome-wide PRS generated using PRS-CS and East Asian summary statistics achieved statistically significant differentiation between cases and controls, especially in the NSCLC and LUAD subtypes. These findings highlight the value of ancestry-matched GWAS inputs and genome-wide modeling approaches in enhancing predictive power.

Interestingly, when using European-based GWAS summary statistics, the performance of PRS-CS was less robust. Although the PRS for SCLC showed nominal significance, overall predictive accuracy was suboptimal, reinforcing the limited transferability of European-derived PRS models to East Asian populations. This observation aligns with previous reports demonstrating that population-specific allele frequencies and linkage disequilibrium structures strongly influence PRS performance.

Notably, PRS-CSx outperformed both single-ancestry PRS models by integrating East Asian and European GWAS data. The model demonstrated improved risk discrimination for overall lung cancer and for NSCLC and LUAD specifically, supporting its utility in cross-ancestry PRS construction. However, despite its superior performance, PRS-CSx did not achieve significant discrimination in LUSC or SCLC subtypes, likely reflecting subtype heterogeneity and limited sample sizes in these groups.

Furthermore, demographic characteristics such as age, sex distribution, and smoking status may also influence PRS performance. These factors can affect both disease risk and the expression of genetic susceptibility, and their imbalance between cases and controls may contribute to variability in model accuracy. For example, smoking, a major environmental risk factor for lung cancer, may interact with genetic risk in subtype-specific ways.

Despite these promising findings, several limitations should be noted. First, the discovery GWAS summary statistics used in this study differ in their availability of subtype-specific information. While the European datasets include partially stratified results by histological subtype, the East Asian GWAS data used in our analyses do not provide subtype-level summary statistics. As our study focuses on a Chinese population, we prioritized East Asian data to ensure ancestry-matching; however, the absence of subtype-specific modeling in these data limits the ability to build optimized PRS for individual lung cancer subtypes, particularly for SCLC and LUSC. The relatively better performance of the European-derived PRS for SCLC may reflect shared genetic architecture between populations, but we cannot exclude the influence of sampling variability or limited power due to small subgroup sizes. Future availability of large-scale, subtype-stratified GWAS in East Asian populations will be critical for improving PRS performance and clarifying the sources of subtype heterogeneity.

In conclusion, our findings emphasize the importance of ancestry-matched GWAS, genome-wide modeling, and cross-ancestry integration for building effective PRS models in non-European populations. While the predictive accuracy remains limited in some lung cancer subtypes, PRS-CSx offers a promising avenue for improved risk stratification in East Asians. These insights highlight the necessity of inclusive genomic research and continued methodological innovation to advance precision medicine in diverse populations.

## Data Availability

The polygenic risk score (PRS) models evaluated in this study were derived from publicly available genome-wide association studies (GWAS), with summary statistics retrieved from previously published datasets (as cited in the manuscript). Due to ethical and privacy concerns, the individual-level genotype and clinical data from the Chinese lung cancer cohort used for evaluation cannot be publicly shared. However, de-identified data supporting the findings of this study are available upon reasonable request from the corresponding author, subject to institutional and ethical approvals.
